# From H_12_C_4_N_2_CdI_4_ to H_11_C_4_N_2_CdI_3_: a highly polarizable CdNI_3_ tetrahedron induced a sharp enhancement of second harmonic generation response and birefringence†

**DOI:** 10.1039/d3sc03052k

**Published:** 2023-08-17

**Authors:** Huai-Yu Wu, Chun-Li Hu, Miao-Bin Xu, Qian-Qian Chen, Nan Ma, Xiao-Ying Huang, Ke-Zhao Du, Jin Chen

**Affiliations:** a College of Chemistry and Materials Science, Fujian Normal University Fuzhou 350002 China cj2015@fjnu.edu.cn; b State Key Laboratory of Structural Chemistry, Fujian Institute of Research on the Structure of Matter, Chinese Academy of Sciences Fuzhou 350002 P. R. China

## Abstract

In this study, we identify a novel class of second-order nonlinear optical (NLO) crystals, non-π-conjugated piperazine (H_10_C_4_N_2_, PIP) metal halides, represented by two centimeter-sized, noncentrosymmetric organic–inorganic metal halides (OIMHs), namely H_12_C_4_N_2_CdI_4_ (*P*2_1_2_1_2_1_) and H_11_C_4_N_2_CdI_3_ (*Cc*). H_12_C_4_N_2_CdI_4_ is the first to be prepared, and its structure contains a CdI_4_ tetrahedron, which led to a poor NLO performance, including a weak and non-phase-matchable second harmonic generation (SHG) response of 0.5 × KH_2_PO_4_ (KDP), a small birefringence of 0.047 @1064 nm and a narrow bandgap of 3.86 eV. Moreover, H_12_C_4_N_2_CdI_4_ is regarded as the model compound, and we further obtain H_11_C_4_N_2_CdI_3_*via* the replacement of CdI_4_ with a highly polarizable CdNI_3_ tetrahedron, which results in a sharp enhancement of SHG response and birefringence. H_11_C_4_N_2_CdI_3_ exhibits a promising NLO performance including 6 × KDP, 4.10 eV, Δ*n* = 0.074 @1064 nm and phase matchability, indicating that it is the first OIMH to simultaneously exhibit strong SHG response (>5 × KDP) and a wide bandgap (>4.0 eV). Our work presents a novel direction for designing high-performance NLO crystals based on organic–inorganic halides and provides important insights into the role of the hybridized tetrahedron in enhancing the SHG response and birefringence.

## Abstract 2

The replacement of CdI_4_ with a CdNI_3_ unit results in H_11_C_4_N_2_CdI_3_ featuring an overwhelming NLO effect (*Cc*, 6 × KDP, Δ*n* = 0.074 @1064 nm, phase matchable) compared to those of H_12_C_4_N_2_CdI_4_ (*P*2_1_2_1_2_1_, 0.5 × KDP, 0.047 @1064 nm, non phase matchable).

## Graphical abstract

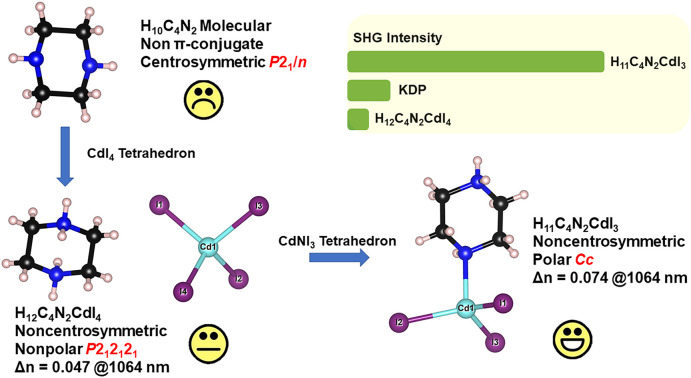

## Introduction

Nonlinear optical (NLO) materials, particularly second harmonic generation (SHG) crystals, offer effective ways for tuning the laser wavelength and expanding its spectral range.^1–6^ Traditionally, high-quality second-order NLO crystals must satisfy several fundamental conditions, such as exhibiting a noncentrosymmetric structure, possessing a strong SHG effect, having a wide bandgap and transmission window, featuring appropriate birefringence for phase matchability, and having a conducive crystal growth method.^7–10^ To date, several commercialized NLO crystals have been used, including KBe_2_BO_3_F_2_ (KBBF), β-BaB_2_O_4_ (BBO), KH_2_PO_4_ (KDP), KTiOPO_4_ (KTP), AgGaS_2_ (AGS), and α-LiIO_3_.

Over the past few years, interest in the development of semi-organic NLO crystals has been steadily increasing. These crystals feature many advantages such as the availability of a wide range of organic components, tunability of inorganic structures, and ease of single crystal growth.^11–15^ Notably, the compounds containing π-conjugated organic moieties have attracted the most attention, and many promising NLO materials in such classes have been reported, such as KLi(HC_3_N_3_O_3_)·2H_2_O (5.3 × KDP),^11^ Lu_5_(C_3_N_3_O_3_)(OH)_12_ (4.2 × KDP),^12^ C(NH_2_)_3_SO_3_F (5 × KDP),^13^ (C_5_H_6_ON)^+^(H_2_PO_4_)^−^ (3 × KDP),^14^ and [*o*-C_5_H_4_NHOH]_2_[I_7_O_18_(OH)]·3H_2_O (8.5 × KDP).^15^ Alongside these compounds, several organic–inorganic metal halides (OIMHs) formed from the combination of a π-conjugated organic cation with a metal-halide polyhedron have also been discovered to show NLO performance, such as (H_7_C_3_N_6_)(H_6_C_3_N_6_)MCl_3_ (M = Hg and Zn; 5 × and 2.8 × KDP),^16,17^ [C_18_H_21_N_4_][AgX_3_]X (X = Cl, Br, and I, 6.2, 6.5 and 7.6 × KDP),^18^ α-(CN_3_H_6_)_3_Cu_2_I_5_ (1.8 × KDP),^19^ and (C_6_H_11_N_2_)PbBr_3_ (8 × KDP).^20^

It is also worth studying the NLO properties of non-π-conjugated OIMHs, which is similar to the approach of expanding the chemical system from π-conjugated borates to non-π-conjugated phosphates or sulfates.^21–27^ Traditionally, the introduction of metal cations with stereo-chemically active lone-pair electrons (SCALP cation, *e.g.* Bi^3+^, Pb^2+^, and Ge^2+^) or d^10^ transition metal cations (d^10^-TM cation, *e.g.* Zn^2+^ and Cd^2+^) can effectively design novel SHG-active non-π-conjugated OIMHs.^28–34^ However, on the one hand, the use of SCLAP cations is known to cause red shifts in absorption edges and reduce bandgaps of the resulting crystals, limiting their potential applications in the ultraviolet (UV) and deep-UV regions.^35–40^ For instance, Mao's group recently reported a series of Ge^2+^-containing OIMHs, in which (CH_3_NH_3_)GeBr_3_ shows a large SHG response (5.3 × KDP) but a small bandgap (2.91 eV). There are also similar cases to C_4_H_9_NOBiBr_7_ (2.2 × urea, 2.67 eV) and (C_6_H_5_(CH_2_)_4_NH_3_)_4_BiI_7_·H_2_O (1.3 × AGS, 2.29 eV).^41^ On the other hand, many non-π-conjugated OIMHs with d^10^-TM cations, such as (C_4_H_10_NO)_2_Cd_2_Cl_6_,^42^ (l/d-C_10_H_20_N_2_O_4_)_2_Cd_5_Cl_12_,^43^l/d-C_6_H_10_N_3_O_2_ZnBr_3_,^44^ [C_5_H_14_NO]CdCl_3_,^45^l/d-C_12_H_20_N_6_O_4_Cd_2_Cl_5_,^44^ and ((CH_3_)_3_NCH_2_Cl)CdCl_3_,^46^ usually possess wide bandgaps (>5.0 eV) but relatively weak SHG effects (<1.0 × KDP). Hence, it is challenging to develop NLO OIMHs that simultaneously exhibit both a strong SHG effect (>5 × KDP) and wide bandgap (>4.0 eV).

To overcome this challenge, our efforts are focused on the non-π-conjugated organic moiety-Cd^2+^/Zn^2+^-I^−^ system. In the beginning, a noncentrosymmetric OIMH, H_12_C_4_N_2_CdI_4_, has been hydrothermally prepared. But our experimental results show that this compound has a small bandgap (3.86 eV) along with a weak and non-phase-matchable SHG response (0.5 × KDP). Such an undesired NLO performance could be attributed to the high symmetry and weak anisotropy of the CdI_4_ tetrahedron. To the best of our knowledge, this phenomenon is reminiscent of metal sulfates based on a tetrahedral SO_4_^2−^ unit. And in metal sulfates, one effective route to overcome this problem is to partially substitute O atoms in SO_4_^2−^, as seen in LiSO_3_F,^47^ Na_10_Cd(NO_3_)_4_(SO_3_S)_4_,^48^ Ba(NH_2_SO_3_)_2_,^49^ Ba(SO_3_CH_3_)_2_,^50^ and so on. Hence, furthermore, H_12_C_4_N_2_CdI_4_ is regarded as the model compound, and we consider that the coordination of H_10_C_4_N_2_ with a Cd^2+^ cation could result in the partial substitution of I^−^ anions in the CdI_4_ tetrahedron, forming a polarizable Cd–I–N unit, which could enhance the SHG response and birefringence in new OIMHs. Additionally, we also hope that new compound involves a smaller number of I^−^ anions in order to exhibit a wider bandgap. Therefore, a novel OIMH, namely H_11_C_4_N_2_CdI_3_, is prepared *via* regulating the synthetic conditions of H_12_C_4_N_2_CdI_4_ (a lower concentration of I^−^ anions in the reaction system). H_11_C_4_N_2_CdI_3_ contains a distorted CdNI_3_ tetrahedron and demonstrates superior NLO performance in comparison to H_12_C_4_N_2_CdI_4_. Specifically, H_11_C_4_N_2_CdI_3_ exhibits a strong and phase-matchable SHG effect (6 × KDP), large birefringence (Δ*n* = 0.062 @1064 nm), and a wide bandgap (4.10 eV), indicating its potential as a promising ultraviolet NLO material.

## Methods

### Materials and synthesis

CdO (>99%), Y_2_O_3_ (>99%), and piperazine (>99%), HI (55–58% wt), were used as purchased from Adamas-beta. There are different synthesis conditions of H_12_C_4_N_3_CdI_4_ and H_11_C_4_N_2_CdI_3_ (Table S1†), and the best conditions (larger size and higher yields) are mentioned as follows. For the preparation of H_12_C_4_N_2_CdI_4_, the starting materials are CdO, (256.8 mg, 2 mmol), piperazine (86.14 mg, 1 mmol), HI (2 mL) and H_2_O (1 mL). A mixture of the starting materials was placed in Teflon pouches (23 mL) sealed in an autoclave which was heated at 90 °C for 72 hours, and cooled to 30 °C at 1.67 °C h^−1^. Yellow block-shaped crystals of H_12_C_4_N_2_CdI_4_ were obtained in high yields of ∼95% (based on Cd). For the preparation of H_11_C_4_N_2_CdI_3_, the starting materials are CdO, (128.4 mg, 1 mmol), Y_2_O_3_, (112.5 mg, 0.5 mmol), piperazine (172.28 mg, 2 mmol), HI (1 mL) and H_2_O (2 mL). A mixture of the starting materials was placed in Teflon pouches (23 mL) sealed in an autoclave which was heated at 110 °C for 72 hours, and cooled to 30 °C at 1.67 °C h^−1^. Yellow block-shaped crystals of H_11_C_4_N_2_CdI_3_ were obtained in high yields of ∼95% (based on Cd).

### Single crystal structure determination

Single-crystal X-ray diffraction data for the title compounds were collected on an Xcalibur, Eos, Gemini diffractometer with Mo Kα radiation (*λ* = 0.71073 Å) at 293 K and a Rigaku XtaLAB Synergy-DW dual-wavelength CCD diffractometer with Cu Kα radiation (*λ* = 1.54184 Å) at 109 K. Data reduction was performed with CrysAlisPro, and absorption correction based on the multi-scan method was applied.^51^ The structures of H_12_C_4_N_2_CdI_4_ and H_11_C_4_N_2_CdI_3_ were determined by the direct methods and refined by full-matrix least-squares fitting on *F*^2^ using SHELXL-2014.^52^ All of the non-hydrogen atoms were refined with anisotropic thermal parameters. The structure was checked for missing symmetry elements using PLATON, and none were found.^53^ The Flack parameters refined for H_12_C_4_N_2_CdI_4_ and H_11_C_4_N_2_CdI_3_ are 0.45(12) and −0.01(2), respectively, indicating the correctness of their absolute structures. In addition, twinning was observed in H_12_C_4_N_2_CdI_4_ and H_11_C_4_N_2_CdI_3_, and the twin laws of these compounds were (−1.0, 0.0, 0.0, 0.0, −1.0, 0.0, 0.0, 0.0, −1.0) and (−1.0, 0.0, 0.0, 0.0, −1.0, 0.0, 0.0, 0.0, −1.0), respectively. Crystallographic data and structural refinements of the compounds are listed in Tables S2–S6.†

### Powder X-ray diffraction

Powder X-ray diffraction (PXRD) patterns were recorded on a Rigaku Ultima IV diffractometer with graphite-monochromated Cu Kα radiation in the 2*θ* range of 10–70° with a step size of 0.02°.

### Thermal analysis

Thermogravimetric analysis (TGA) was performed with a Rigaku TG-DTA 8121 unit under an Ar atmosphere, at a heating rate of 10 °C min^−1^ in the range from 30 to 800 °C.

### Optical measurements

The infrared (IR) spectrum was recorded on a Thermo Fisher Nicolet 5700 FT-IR spectrometer in the form of KBr pellets in the range from 4000 to 400 cm^−1^.

The ultraviolet-visible-infrared (UV-vis-IR) spectrum in the range of 200–800 nm was recorded on a PerkinElmer Lambda 750 UV-vis-NIR spectrophotometer. The reflectance spectrum was converted into an absorption spectrum by using the Kubelka–Munk function.^54^

### Second harmonic generation measurements

Powder SHG measurements were carried out with a *Q* switch Nd:YAG laser generating radiation at 1064 nm according to the method of Kurtz and Perry.^55^ Crystalline H_12_C_4_N_2_CdI_4_ and H_11_C_4_N_2_CdI_3_ samples were sieved into distinct particle-size ranges (50–70, 70–100, 100–140, 140–200, 200–250 and 250–325 μm). Sieved KH_2_PO_4_ (KDP) samples in the same particle-size ranges were used as references.

### Elemental analysis

The elemental content was measured on a Vario EL Cube elemental analyzer from Elementar Analysensysteme GmbH, Germany. The combustion temperature was 800 °C (Table S7†).

### Energy-dispersive X-ray spectroscopy

Microprobe elemental analyses were performed and the elemental distribution maps were obtained on a field-emission scanning electron microscope (Phenom LE) equipped with an energy-dispersive X-ray spectroscope (EDS, Phenom LE).

### Computational method

The electronic structures and optical properties were obtained using a plane-wave pseudopotential method within density functional theory (DFT) implemented in the total energy code CASTEP.^56,57^ For the exchange and correlation functional, we chose Perdew–Burke–Ernzerhof (PBE) in the generalized gradient approximation (GGA).^58^ The interactions between the ionic cores and the electrons were described by the norm-conserving pseudopotential.^59^ The following orbital electrons were treated as valence electrons: I-5s^2^5p^5^, Cd-4s24p^6^5s^2^, H-1s^1^, C-2s^2^2p^2^ and N-2s^2^2p^3^. The numbers of plane waves included in the basis sets were determined at a cutoff energy of 700 eV. During the SCF and optical-property calculations of the two compounds, the *k*-point separation was set to 0.04 Å^−1^ to perform the numerical integration of the Brillouin zone, and the corresponding *k*-point samplings are 3 × 2 × 2 and 2 × 4 × 2 for H_12_C_4_N_2_CdI_4_ and H_11_C_4_N_2_CdI_3_, respectively. After the principal axis transformation, the *k*-point sampling of H_11_C_4_N_2_CdI_3_ changes to 2 × 3 × 2 when the *k*-point separation is 0.04 Å^−1^. The other parameters and convergent criteria were the default values of the CASTEP code.^56,57^

The calculations of second-order NLO susceptibilities were based on length-gauge formalism within the independent particle approximation.^60,61^ The second-order NLO susceptibility can be expressed as*χ*_*abc*_*L*(−2*ω*; *ω*, *ω*) = *χ*_*abc*_ inter (−2*ω*; *ω*, *ω*) + *χ*_*abc*_ inter(−2*ω*; *ω*, *ω*) + *χ*_*abc*_ mod(−2*ω*; *ω*, *ω*)where the subscript *L* denotes the length gauge, and *χ*_*abc*_ inter, *χ*_*abc*_ intra and *χ*_*abc*_ mod give the contributions to *χ*_*abc*_*L* from interband processes, intraband processes, and the modulation of interband terms by intraband terms, respectively.

The convergence test of the SHG coefficient upon *k*-point sampling and empty bands with H_11_C_4_N_2_CdI_3_ as an example was carried out (Table S9†), and we found that the SHG coefficient gradually converges when the *k*-point separation is not more than 0.04 Å^−1^ (2 × 3 × 2) and the quantity of the empty bands is not less than 2 times that of valence bands (280 bands). So the choice of *k*-point sampling and the empty band number during the optical property calculation is reasonable.

## Results and discussion

In order to investigate the differences in the synthesis conditions of H_12_C_4_N_3_CdI_4_ and H_11_C_4_N_2_CdI_3_, we conducted multiple sets of control experiments as shown in Table S1.† Several observations were made: (1) the molar ratio of Cd to H_10_C_4_N_2_ had no impact on whether H_12_C_4_N_3_CdI_4_ or H_11_C_4_N_2_CdI_3_ was produced as the final product; (2) changes in temperature had minimal effects on the synthesis of H_12_C_4_N_3_CdI_4_; (3) the ratio of HI to H_2_O was the determining factor in the preparation of H_12_C_4_N_3_CdI_4_ or H_11_C_4_N_2_CdI_3_. We hypothesize that a lower HI : H_2_O ratio can reduce the concentration of iodide ions in the reaction system, thus facilitating direct coordination bonding between Cd^2+^ cations and H_10_C_4_N_2_, resulting in the formation of H_11_C_4_N_2_CdI_3_; (4) in the preparation process of H_11_C_4_N_2_CdI_3_, Y_2_O_3_ is essential and cannot be omitted. However, indeed, the specific role of Y_2_O_3_ in the synthesis process of H_11_C_4_N_2_CdI_3_ is currently unclear due to limitations in our current technological means. Their measured powder XRD patterns are consistent with those of simulated data, confirming the phase purity (Fig. S1†). The EDS results of the Cd : I ratios are 1.0 : 4.10 and 1.0 : 2.97 for H_12_C_4_N_2_CdI_4_ and H_11_C_4_N_2_CdI_3_, respectively (Fig. S2†). In addition, elemental analysis of C, N, and H atoms is provided in Table S6† with weight%: C 6.77, H 1.63, and N 4.02 for H_12_C_4_N_2_CdI_4_; C 8.30, H 1.85, and N 4.53 for H_11_C_4_N_2_CdI_3_ (Table S7†). Both of them are in good agreement with the crystal structure solution.

### Crystal structure

**Fig. 1:**
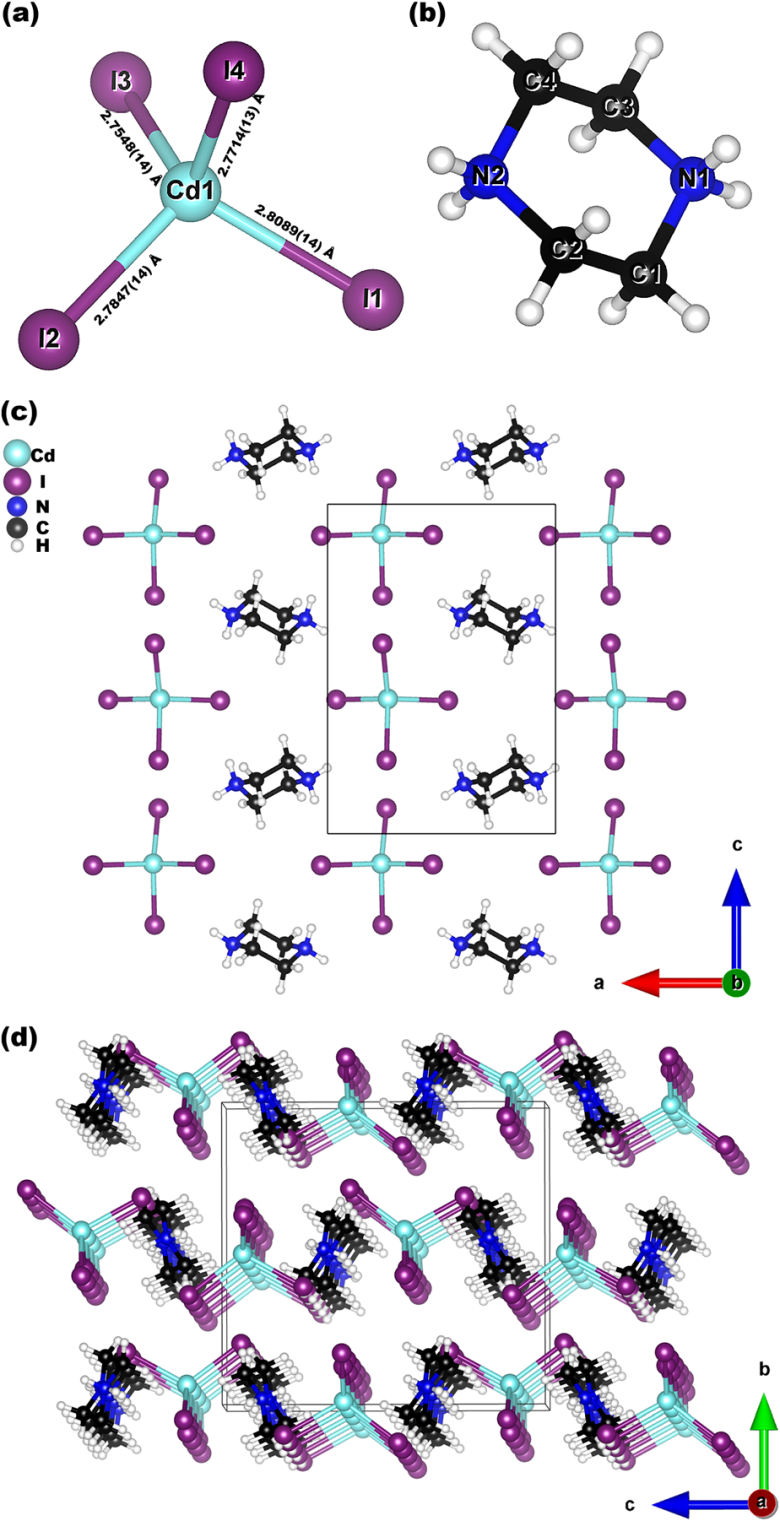
Views of the structures of the CdI_4_ tetrahedron (a), the chair shaped H_2_PIP^2+^ cation (b), the quasi-2D H_12_C_2_N_4_CdI_4_ layer along the *b*-axis (c) and H_12_C_2_N_4_CdI_4_ along the *a*-axis (d).

H_1_2C_4_N_2_CdI_4_ crystallizes in a noncentrosymmetric space group *P*2_1_2_1_2_1_ (No. 19) with one cationic H_12_C_2_N_4_^2+^ (H_2_PIP^2+^) six-membered ring and one anionic CdI_4_^2−^ tetrahedron in each asymmetric unit (Fig. 1). In the H_12_C_4_N_2_-ring, each C and N atom exhibits sp^3^ hybridization, with C–N/C–C distances of 1.49(2)–1.50(2) Å and 1.474(17)–1.496(18) Å, respectively. The bond angles in the H_12_C_4_N_2_-ring range from 110.5(12)–111.2(11)°, which is consistent with those of previously reported piperazine compounds.^62,63^ As a result, the H_12_C_2_N_4_^2+^ cation takes on a chair conformation, rather than a planar π-conjugated structure. Each Cd^2+^ cation bonds with four I^−^ anions to form a CdI_4_ tetrahedron, with Cd–I distances ranging from 2.7548(14)–2.8089(14) Å and I–Cd–I angles of 104.50(4)–113.75(5)°. Neighboring H_12_C_2_N_4_ and CdI_4_ groups are isolated from each other and arranged in a quasi-two-dimensional (quasi-2D) [H_12_C_4_N_2_CdI_4_] layer (Fig. 1c), parallel to the *ac* plane. These pseudo-layers stack along the *b* direction to form the entire 3D network of H_12_C_4_N_2_CdI_4_ (Fig. 1d).

**Fig. 2:**
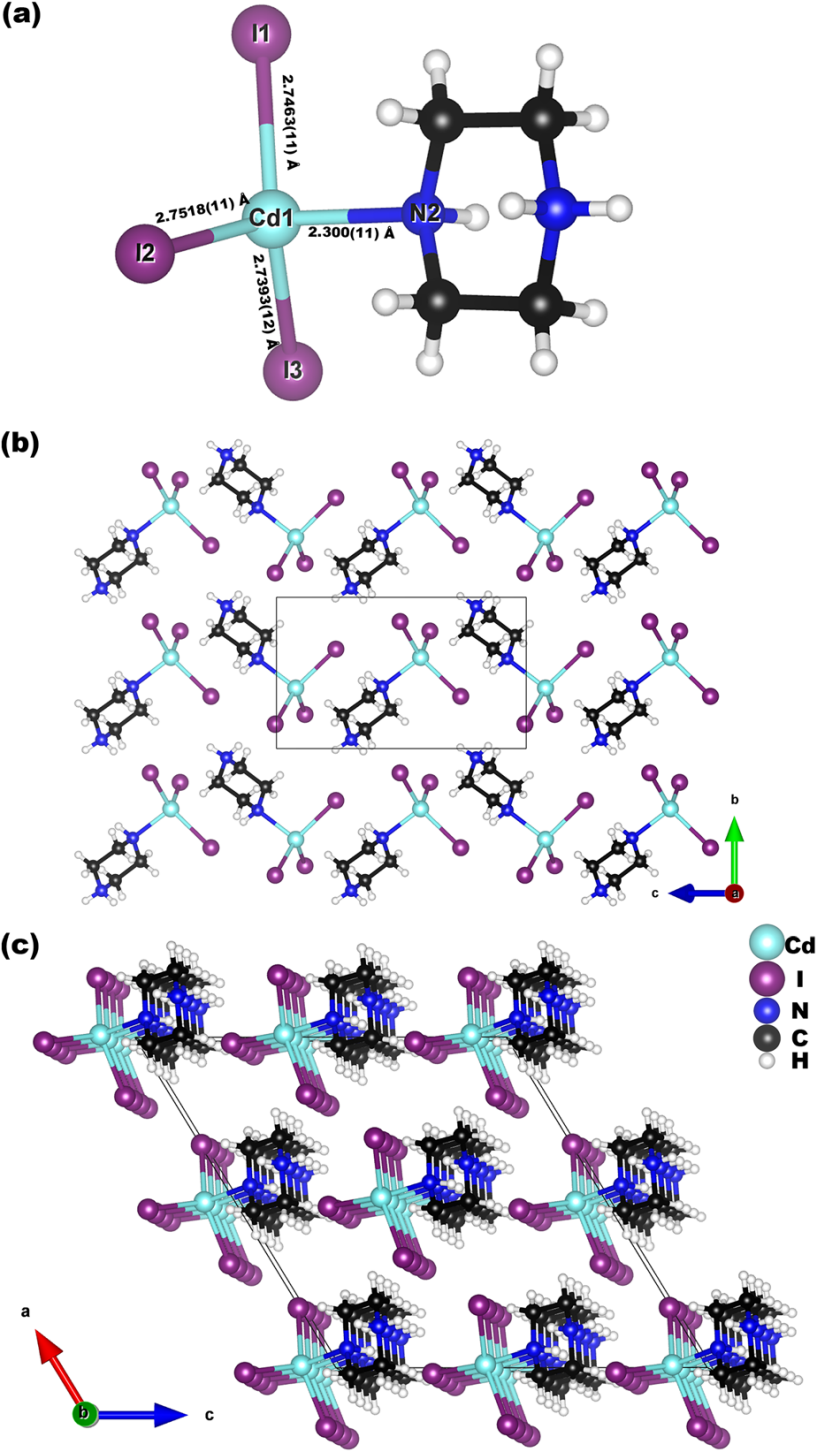
Views of the structures of the H_11_C_4_N_2_CdI_3_ molecule (a), the quasi-2D H_11_C_4_N_2_CdI_3_ layer along the *a*-axis (b) and H_11_C_4_N_2_CdI_3_ along the *b*-axis.

H_11_C_4_N_2_CdI_3_ crystallizes in the polar space group *Cc* (No. 9), with an asymmetric unit including one protonated-piperazine group, one Cd, and three I atoms (Fig. 2a). In the chair-shaped H_11_C_4_N_2_-ring, C–N and C–C distances are 1.49(2)–1.532(17) Å and 1.462(17)–1.509(19) Å, respectively, and the bond angles are in the range of 108.7(11)–114.5(9)°, which are consistent with those of previously reported piperazine compounds.^62,63^ Each Cd^2+^ cation connects with one N atom and three I atoms to form a *N*-hybrid polyhedron, a CdNI_3_ tetrahedron. The length of Cd–N (2.300(11) Å) is smaller than those of Cd–I (2.7393(12)–2.7518(11) Å), and the bond angles of N–Cd–I and I–Cd–I are 100.8(3)–104.0(3)° and 112.80(3)–117.07(4)°, respectively. Furthermore, one H_11_C_4_N_2_-ring is connected with one CdNI_3_ tetrahedron *via* the corner-sharing of N atoms, forming a H_11_C_4_N_2_CdI_3_ molecule (Fig. 2a). Neighboring H_11_C_4_N_2_CdI_3_ molecules are interconnected in a quasi-1D zigzag chain along the *c*-direction, which is further arranged in quasi-2D layers parallel to the *bc* plane (Fig. 2b). These quasi-2D layers are stacked upward along the *a*-axis, resulting in the structure of H_11_C_4_N_2_CdI_3_ (Fig. 2c).

### Structural comparison

It is interesting to compare the structural differences of the title compounds to further understand why H_11_C_4_N_2_CdI_3_ is a polar compound while H_12_C_4_N_2_CdI_4_ is not. Firstly, we believe that the distinct coordination environments of Cd^2+^ ions play a key role. In H_12_C_4_N_2_CdI_4_, the molar ratio of Cd to I is 4, which results in each Cd^2+^ ion being surrounded by only four I atoms, forming a nearly undistorted CdI_4_ tetrahedron. In contrast, in H_11_C_4_N_2_CdI_3_, Cd: I is 3, which enables the N atom on the H_10_C_4_N_2_ unit to participate in Cd^2+^ coordination, forming a distorted CdNI_3_ tetrahedron. Secondly, the calculated dipole moments of the CdI_4_ and CdNI_3_ units are 3.27 and 5.94 *D* (Table S10†), respectively, indicating that CdNI_3_ exhibits more anisotropy, favoring the formation of a polar structure. Finally, the arrangement of Cd^2+^-centered tetrahedra can also affect the polarity. In H_12_C_4_N_2_CdI_4_, each H_12_C_4_N_2_^2+^ cation is surrounded by four CdI_4_ tetrahedra, and their dipole moments cancel out each other along the *a*, *b*, and *c* axes (Table S10†). As a result, although H_12_C_4_N_2_CdI_4_ has a noncentrosymmetric structure, it is nonpolar, and its net dipole moment in a unit cell is zero. However, in H_11_C_4_N_2_CdI_3_, the dipole moments of CdNI_3_ tetrahedra cancel out each other only along the *b* axis, while they add up along the *a* and *c* axes, resulting in a large net dipole moment of about 17.09 *D* (Table S10†). Therefore, H_11_C_4_N_2_CdI_3_ is a noncentrosymmetric and polar compound.

### Thermal and optical properties

**Fig. 3:**
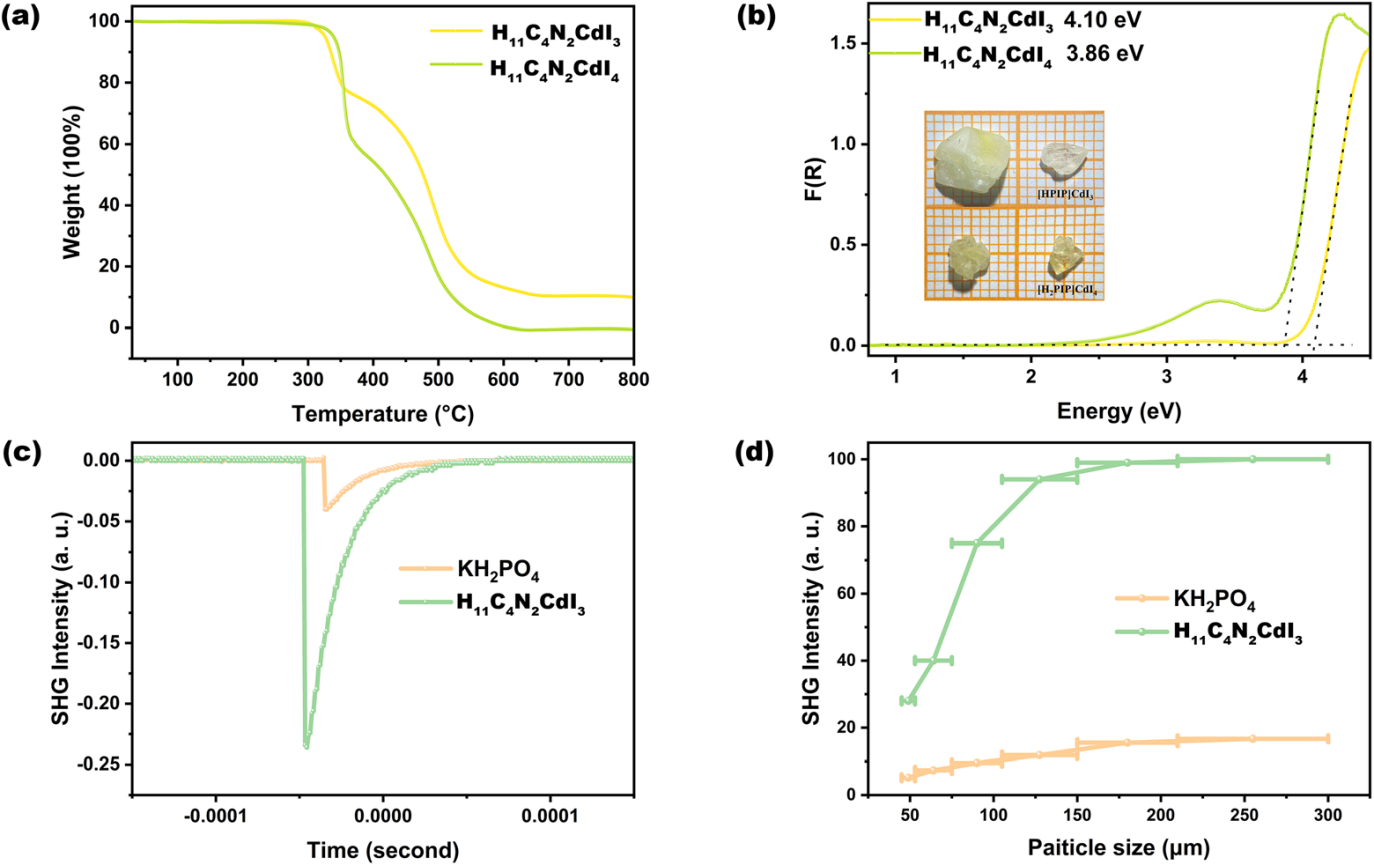
TGA curves (a), UV-vis diffuse reflectance spectra (inset picture is the crystal photo) (b), measured oscilloscope traces of the SHG signals (150–210 mm) (c) and SHG intensity *vs.* particle size of compounds under 1064 nm laser radiation (d). KDP served as the reference.

Thermogravimetric analysis (TGA) shows the good thermal stabilities of H_12_C_4_N_2_CdI_4_ and H_11_C_4_N_2_CdI_3_, at around 300 °C (Fig. 3a). These values are higher than those of previously reported NLO organic–inorganic halides, including (H_7_C_3_N_6_)(H_6_C_3_N_6_)HgCl_3_ (225 °C),^16^ (CH_3_NH_3_)GeBr_3_ (500 K),^30^, [C_18_H_21_N_4_][AgI_3_]I (260 °C)^18^ and Rb_2_[PbI_2_(HCOO)_2_] (240 °C).^64^ The UV-vis spectra show the absorption edges of 291 and 289 nm for H_12_C_4_N_2_CdI_4_ and H_11_C_4_N_2_CdI_3_, respectively (Fig. S4†). Furthermore, compared to H_12_C_4_N_2_CdI_4_, H_11_C_4_N_2_CdI_3_ exhibits a slightly larger bandgap (4.10 eV *vs.* 3.86 eV) (Fig. 3b), which can primarily be attributed to a reduction in the number of I^−^ ions. Besides, the bandgaps of H_12_C_4_N_2_CdI_4_ and H_11_C_4_N_2_CdI_3_ are wider than that of the NLO *o*-containing SCALP cation, such as (C_4_H_10_NO)PbCl_3_ (3.55 eV),^65^ (C_7_H_15_NCl)SbCl_4_ (3.05 eV),^66^ (C_6_H_5_(CH_2_)_4_NH_3_)_4_BiI_7_·H_2_O (2.29 eV),^41^ Rb_2_[PbI_2_(HCOO)_2_] (240 °C) (3.40 eV),^64^, (R/S-C_16_H_22_N_2_Cl)_2_SnCl_6_ (3.39 eV)^67^ and (CH_3_NH_3_)GeBr_3_ (2.91 eV).^30^

### SHG properties

Since both H_12_C_4_N_2_CdI_4_ and H_11_C_4_N_2_CdI_3_ are noncentrosymmetric, their SHG effects are measured according to the method of Kurtz and Perry.^55^ For H_12_C_4_N_2_CdI_4_, as shown in Fig. S5,† the measurement result reveals unwanted non-phase matchable NLO properties, in which the SHG intensity of the crystalline samples exhibits an initial increase, followed by a subsequent decrease as the particle size increased. The maximum value, which is approximately 90 μm, is observed to be about 0.5 times that of KH_2_PO_4_ (KDP). This phenomenon is reminiscent of the behavior exhibited by traditional phosphate and sulfate compounds, where the CdI_4_ tetrahedron exhibits minimal distortion, and its optical anisotropy is mutually canceled within the *P*2_1_2_1_2_1_ space group. Conversely, as shown in Fig. 3c and d, the SHG intensity gradually increased and saturated with increasing particle size, indicating that H_11_C_4_N_2_CdI_3_ exhibits phase-matching NLO properties. The SHG effect of H_11_C_4_N_2_CdI_3_ is about six times that of KDP, measured at a particle size of 150–210 μm.

It is interesting to investigate the structure–property relationship among the title compounds and formerly reported Cd-centered NLO OIMHs, such as [(CH_3_)_3_NCH_2_Cl]CdCl_3_,^46^ (H_10_C_4_NO)_2_Cd_2_Cl_6_,^42^ (l/d-Hpro)_2_Cd_5_Cl_12_,^43^ (l/d-Hpro)(l-pro)CdCl_3_,^43^ and l/d-C_12_H_20_N_6_O_4_Cd_2_Cl_5_.^44^ The structure of [(CH_3_)_3_NCH_2_Cl]CdCl_3_ contains a 0D CdCl_6_ octahedron, whereas in (l/d-Hpro)_2_Cd_5_Cl_12_ and (l/d-Hpro)(l-pro)CdCl_3_, the CdCl_6_ octahedra are inter-connected into the 2D Cd_5_Cl_12_ layer *via* edge-sharing and the 1D CdCl_3_ chain *via* face-sharing, respectively. Notably, such CdCl_6_ octahedra only exhibit low optical anisotropy, with very small dipole moments (<0.1 *D*). Hence, their SHG response is relatively weak (<1.0 × KDP). Besides, the N/O atoms in non-π-conjugated organic groups are sp^3^ hybridized, which makes them more likely to form coordination bonds with Cd^2+^ cations in non-π-conjugated OIMHs. Therefore, many of them feature an *O*/*N*-hybridized polyhedron. In (H_10_C_4_NO)_2_Cd_2_Cl_6_, neighbouring CdCl_6_ and CdCl_4_O_2_ octahedra are bridged into a 1D chain *via* edge-sharing. l/d-C_12_H_20_N_6_O_4_Cd_2_Cl_5_ contains a CdOCl_4_ trigonal bipyramid, and each pair of them is formed into a Cd_2_O_2_Cl_6_ dimer *via* edge-sharing. Importantly, these Cd–O–Cl polyhedra feature larger dipole moments (1.14–5.03 *D*) that a CdCl_6_ octahedron. However, for example, the undesired arrangement of CdOCl_4_ groups leads to a small net dipole moment (1.19–1.20 *D*) and weak SHG response in l/d-C_12_H_20_N_6_O_4_Cd_2_Cl_5_ (∼0.2 × KDP). Most importantly, in this work, H_12_C_4_N_2_CdI_4_ and H_11_C_4_N_2_CdI_3_ involve CdI_4_ (3.27 *D*) and CdNI_3_ (5.94 *D*) tetrahedra, respectively, in which the polarization magnitudes of CdNI_3_ tetrahedra are mutually superimposed along the *a*- and *c*-axes resulting in a large net dipole moment of H_11_C_4_N_2_CdI_3_ (17.09 *D*) (Table S10†). Accordingly, compared with previously reported Cd-centered OIMHs and H_12_C_4_N_2_CdI_4_, H_11_C_4_N_2_CdI_3_ features a far large SHG response (6 × KDP).

**Fig. 4:**
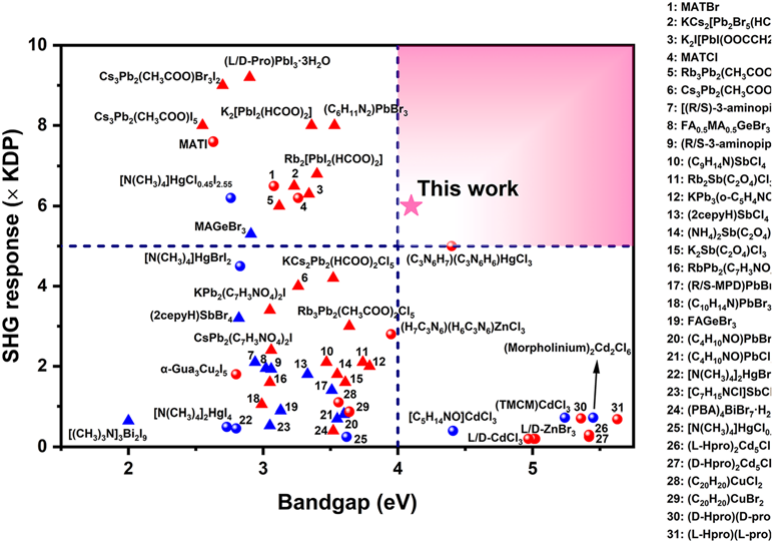
SHG response and energy bandgap of representative NLO organic–inorganic halides. Red ball: compound containing both a π-conjugated organic group and SCALP cation, red triangle: compound containing a π-conjugated organic group but without a SCALP cation, blue ball: compound without a π-conjugated organic group but with a SCALP cation, blue triangle: other compounds.

Besides, importantly, compared with recently reported NLO OIMHs with SCALP or other d^10^-TM cations such as (C_4_H_10_NO)PbBr_3_ (0.81 × KDP),^65^ [C(NH_2_)_3_]SbF_4_ (2 × KDP),^32^ [N(CH_3_)_4_]HgBrI_2_ (4.5 × KDP),^68^ (H_7_C_3_N_6_)(H_6_C_3_N_6_)HgCl_3_ (5.8 × KDP),^16^ α-(CN_3_H_6_)_3_Cu_2_I_5_ (1.8 × KDP),^19^ (CH_3_NH_3_)GeBr_3_ (5.3 × KDP)^30^ and (C_20_H_20_P)CuCl_2_ (1.1 × KDP),^69^ H_11_C_4_N_2_CdI_3_ has a stronger SHG response (6 × KDP). Furthermore, the SHG responses and bandgaps of selected organic–inorganic halides are summarized in Fig. 4 and Table S11† (the relevant references are provided in the ESI†). Unlike other NLO crystals, H_11_C_4_N_2_CdI_3_ simultaneously exhibits both a strong SHG effect (>5 × KDP) and wide bandgap (>4.0 eV), which makes it promising as a high-performance ultraviolet NLO crystal.

### Structure–property relationship analysis

**Fig. 5:**
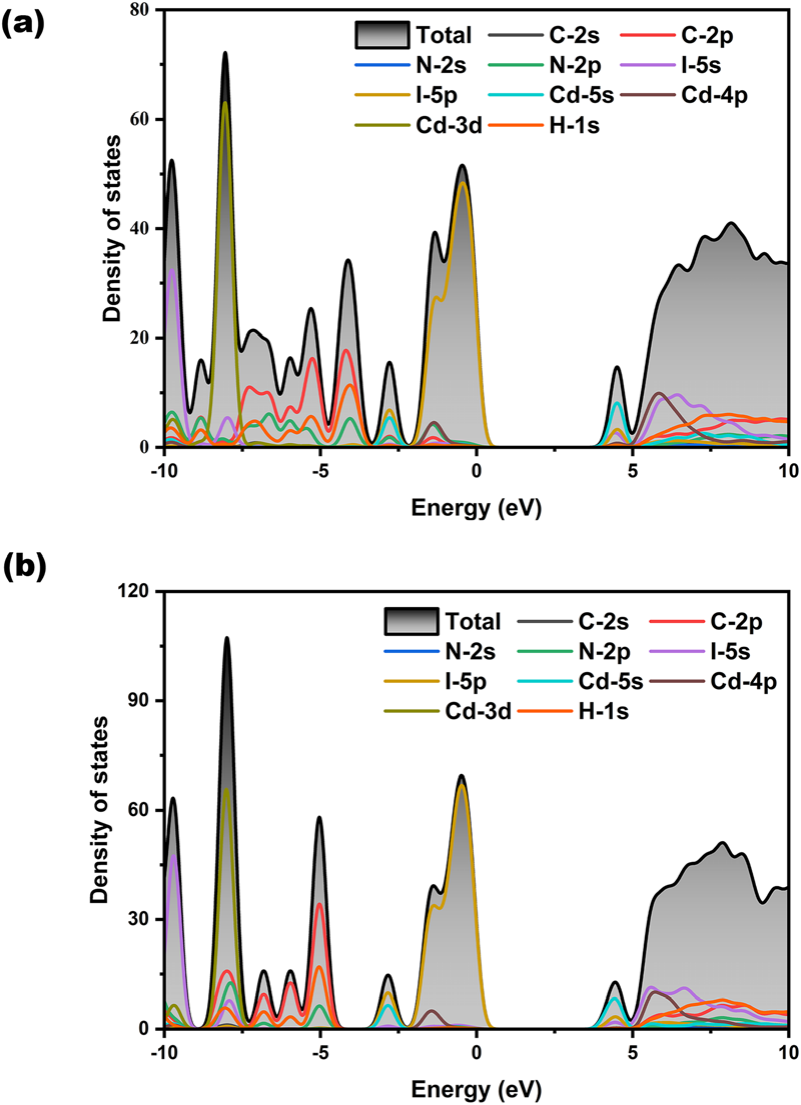
The density of states of H_12_C_4_N_2_CdI_4_ and H_11_C_4_N_2_CdI_3_ (b).

The calculated bandgaps of H_12_C_4_N_2_CdI_4_ and H_11_C_4_N_2_CdI_3_ are provided in Fig. S6,† indicating that they are direct bandgap compounds with values of 3.895 and 4.102 eV for H_12_C_4_N_2_CdI_4_ and H_11_C_4_N_2_CdI_3_, respectively, being comparable to the results from measurement (3.86 and 4.10 eV). Partial density of states (PDOS) of H_12_C_4_N_2_CdI_4_ and H_11_C_4_N_2_CdI_3_ is presented in Fig. 5a and b, respectively. The PDOS plots of both compounds are similar. The contribution to the valence band maximum (VBM) mainly comes from the I-p orbitals, as well as a small portion of the Cd-p orbitals. For the conduction band minimum (CBM), the contribution sizes rank in the order of Cd-s, I-p, and I-s orbitals. It is worth noting that in H_11_C_4_N_2_CdI_3_, a small amount of N-p orbitals also contributes to the VBM, which is consistent with the existence of the Cd–N bond. Therefore, we believe that the bandgaps of H_12_C_4_N_2_CdI_4_ and H_11_C_4_N_2_CdI_3_ are determined by the Cd-centered tetrahedra.

**Fig. 6:**
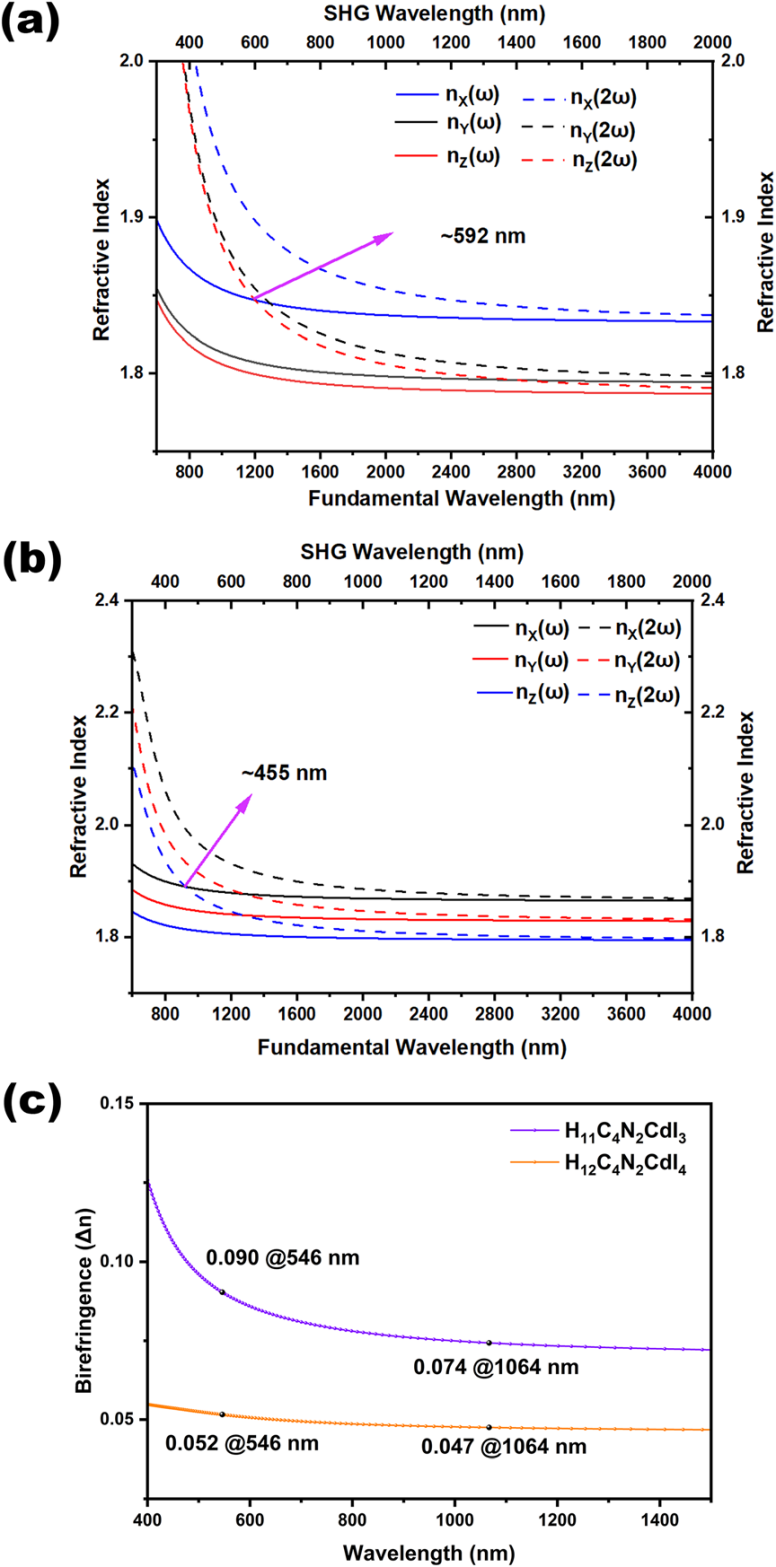
Refractive index dispersion curves for fundamental and second-harmonic light of H_12_C_4_N_2_CdI_4_ (a) and H_11_C_4_N_2_CdI_3_ (b), as well as the calculated birefringence (c). The type-I phase matching wavelengths in different planes were evaluated based on the calculated refractive index, in which we consider the type-I phase matching condition of *n*(*ω*) = *n*(2*ω*).

Herein, we use the calculated birefringence (Δ*n*) and phase matching wavelength based on the relations between the wavelength and refractive index under both fundamental and harmonic conditions (Fig. 6). Both of them are biaxial crystals with three principal optical axes, *i.e.*, *X*, *Y*, and *Z*. For the orthorhombic crystal H_12_C_4_N_2_CdI_4_, the calculated refractive index curves after structure optimization show the order of *n*_*c*_ > *n*_*a*_ > *n*_*b*_ in the low-frequency range, and to satisfy the biaxial condition *n*_*X*_ > *n*_*Y*_ > *n*_*Z*_, the transformation of *c* → _*X*_, *a* → _*Y*_, and *b* → _*Z*_ is performed and a correct order of *n*_*X*_ > *n*_*Y*_ > *n*_*Z*_ is shown in Fig. 6a. For the monoclinic crystal H_11_C_4_N_2_CdI_3_, the crystallographic axes and principal dielectric axes are not consistent with each other in the *ac* plane, so the principal dielectric axes in the *ac* plane must be determined after structure optimization and before the optical calculation. According to the principal axis transformation method in our previous work, the rotation angle *θ* between the original coordinate axes (*i.e. y*∥*b*, *z*∥*c*) and the principal dielectric axes is found to be 25.179°.^70^ After the principal axis transformation and based on the principal dielectric coordinate system, the calculated refractive index curves show an order of *n*_*X*_ > *n*_*Y*_ > *n*_*Z*_ and satisfy the biaxial condition. The shortest wavelengths for type-I phase matching are approximately 592 nm and 466 nm for H_12_C_4_N_2_CdI_4_ and H_11_C_4_N_2_CdI_3_, respectively. This implies that under 1064 nm laser radiation, H_11_C_4_N_2_CdI_3_ can achieve type-I phase matching, while H_12_C_4_N_2_CdI_4_ cannot. These findings are consistent with our SHG measurements. These calculated birefringences have been obtained from the refractive index difference (*n*_*X*_ – *n*_*Z*_) (Fig. 6c), in which H_11_C_4_N_2_CdI_3_ possesses a larger birefringence of (0.090 @546 nm, 0.074 @1064 nm) than that of H_12_C_4_N_2_CdI_4_ (0.052 @546 nm, 0.047 @1064 nm). Since piperazine is a non-π conjugated group with negligible contribution of birefringence, the larger birefringence of H_11_C_4_N_2_CdI_3_ can be attributed to the microscopic asymmetry and well-arranged structure of the CdNI_3_ group, exemplified by the dipole moment calculation (Table S10†). Consequently, from H_12_C_4_N_2_CdI_4_ to H_11_C_4_N_2_CdI_3_, the polarizable CdNI_3_ tetrahedron induces an enlarged birefringence, ensuring phase-matching performance of H_11_C_4_N_2_CdI_3_.

**Fig. 7:**
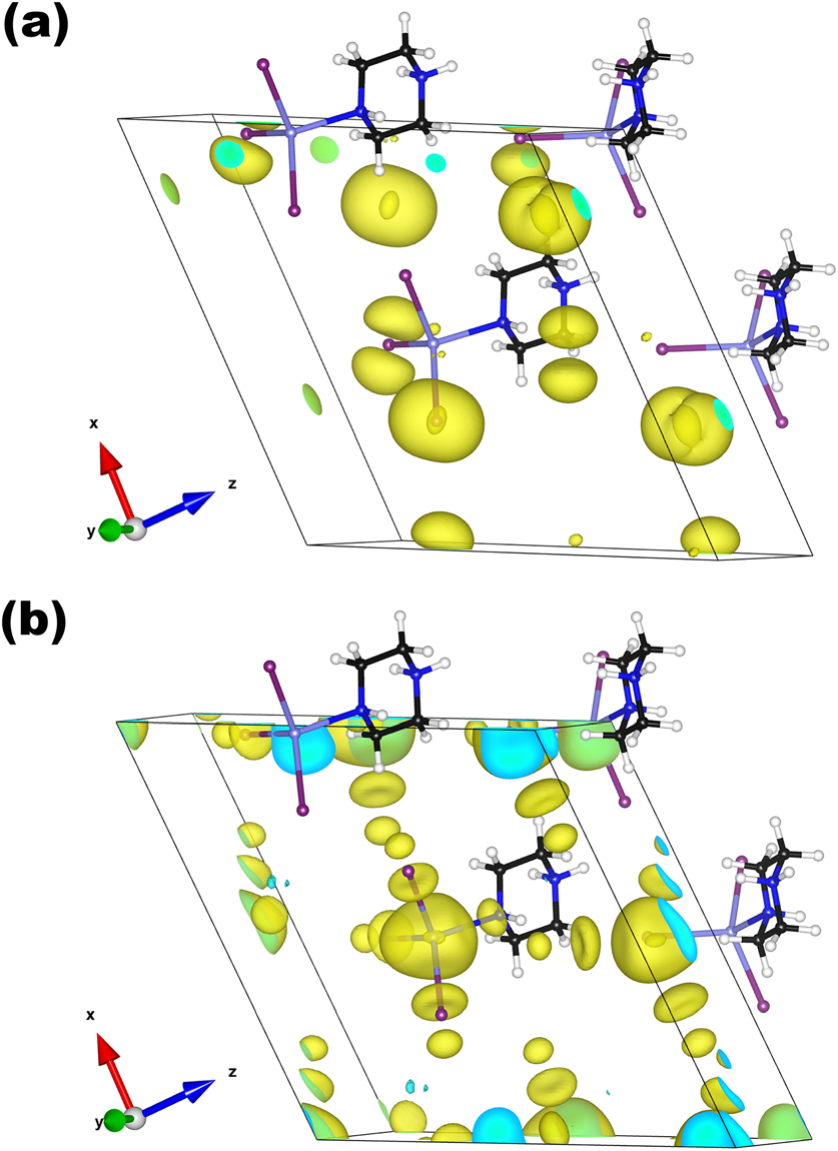
SHG density plots for H_11_C_4_N_2_CdI_3_: VB (a) and CB (b).

We further calculate the theoretical SHG coefficients of H_12_C_4_N_2_CdI_4_ and H_11_C_4_N_2_CdI_3_, as shown in Table S12.† For H_12_C_4_N_2_CdI_4_, it has three non-zero independent SHG tensors with the same value of 0.12 pm V^−1^, which is about 0.3 times that of KDP (0.39 pm V^−1^), being consistent with the experimental value (0.5 × KDP). More importantly, for H_11_C_4_N_2_CdI_3_, its largest independent SHG tensor is *d*_11_ = 2.74 pm V^−1^, about 7 times larger than that of KDP, which is agreement with the experimental result (6 × KDP). To explore the origin of the better SHG effect of H_11_C_4_N_2_CdI_3_, we further calculated the SHG density distribution of the *d*_11_ tensor (Fig. 7). In the VB, the main source of the SHG effect is the I-5p states. In addition, there are small contributions from the Cd 5s states and the N-2p states from the Cd–N bond. The maximum source in the CB is the Cd-5s orbitals, followed by the I-5s orbitals and the N-2p orbitals in the Cd–N bond. Furthermore, the contributions of each unit to the SHG response are calculated, and the CdNI_3_ tetrahedron has a contribution as high as 97.96%. Therefore, we believe that the distorted CdNI_3_ tetrahedron is the primary origin of the outstanding SHG effect of H_11_C_4_N_2_CdI_3_.

## Conclusions

In summary, we have discovered two new organic–inorganic metal halides (OIMHs), H_12_C_4_N_2_CdI_4_ and H_11_C_4_N_2_CdI_3_, by combining the protonated pyrazine cation with d^10^-TM-halide tetrahedra. The NLO properties of non-π-conjugated piperazine compounds are studied for the first time. Despite being a noncentrosymmetric compound, H_12_C_4_N_2_CdI_4_ exhibits a weak and non-phase-matchable SHG effect. In contrast, H_11_C_4_N_2_CdI_3_ is a polar compound with excellent NLO performance, including a strong SHG response (6 × KDP), a wide bandgap (4.10 eV), large birefringence (0.074 @1064 nm) for phase-matching requirement, good thermal stability (300 °C), and a suitable crystal growth habit. The structure–property relationship analysis indicates that the highly polarizable *N*-hybridized CdNI_3_ tetrahedron in the H_11_C_4_N_2_CdI_3_ structure is the primary cause of the birefringence and SHG effect. Our work provides new insights for exploring semi-organic NLO crystals that do not contain π-conjugated organic components. Further exploration of OIMHs containing *N*- or *O*-hybridized metal-halide polyhedra is currently underway.

## Data availability

The experimental or computational data can be obtained *via* contacting the corresponding author.

## Author contributions

Wu Huai-Yu: conceptualization, methodology, writing – original draft, data curation , visualization; Xu Miao-Bin, Chen Qian-Qian, and Ma Nan: data curation; Hu Chun-Li: formal analysis; Huang Xiao-Ying, Du Ke-Zhao and Chen Jin: writing – review & editing, supervision.

## Conflicts of interest

There are no conflicts to declare.

## Supplementary Material

SC-014-D3SC03052K-s001

SC-014-D3SC03052K-s002

SC-014-D3SC03052K-s003

SC-014-D3SC03052K-s004

SC-014-D3SC03052K-s005
