# Supplementary material for: From H12C4N2CdI4 to H11C4N2CdI3: a highly polarizable CdNI3 tetrahedron induced a sharp enhancement of second harmonic generation response and birefringence
Source: Chem Sci. 2023 Aug 17;14(35):9533–42. doi: 10.1039/d3sc03052k (PMC10498671; doi:10.1039/d3sc03052k)
Supplement: SC-014-D3SC03052K-s004 [file SC-014-D3SC03052K-s004.pdf]

Electronic Supplementary Material (ESI) for Chemical Science.
This journal is © The Royal Society of Chemistry 2023

From $H_{12}C_4N_2CdI_4$ to $H_{11}C_4N_2CdI_3$: highly polarizable $CdNI_3$ tetrahedron induced a shape enhancement of second harmonic generation response and birefringence

Huai-Yu Wu,[a] Chun-Li Hu,[b*] Miao-Bin Xu,[a] Qian-Qian Chen,[b] Nan Ma,[b] Xiao-Ying Huang,[b] Ke-Zhao Du[a*] and Jin Chen[a,b*]

a: College of Chemistry and Materials Science, Fujian Normal University, Fuzhou, 350002 China. Email: cj2015@fjnu.edu.cn

b: State Key Laboratory of Structural Chemistry, Fujian Institute of Research on the Structure of Matter, Chinese Academy of Sciences, Fuzhou, 350002, P. R. China.

Table S1. The synthesis conditions of $H_{12}C_4N_3CdI_4$ and $H_{11}C_4N_2CdI_3$.

NO.	Starting Materials (mmol)			HI (mL)	H_2O (mL)	Reaction Temperature (°C)	Product
	CdO	$H_{10}C_4N_2$	Y_2O_3				
1.	2	1	0	2	1	90	$H_{12}C_4N_2CdI_4$
2.	1	2	0	2	1		
3.	2	1	0	3	1		
4.	1	2	0	3	1		
5.	2	1	0	2	1	110	$H_{12}C_4N_2CdI_4$
6.	1	2	0	2	1		
7.	2	1	0	3	1		
8.	1	2	0	3	1		
9.	2	1	0.5	2	1	110	$H_{12}C_4N_2CdI_4$
10.	1	2	0.5	2	1		
11.	2	1	0.5	3	1		
12.	1	2	0.5	3	1		
13.	2	1	0	1	2	110	None
14.	1	2	0	1	2		
15.	2	1	0	0.5	2		
16.	1	2	0	0.5	2		
17.	2	1	0.5	1	2	110	$H_{11}C_4N_2CdI_3$
18.	1	2	0.5	1	2		
19.	2	1	0.5	0.5	2	110	$H_{11}C_4N_2CdI_3$
20.	1	2	0.5	0.5	2		

Table S2. Crystallographic data for $H_{12}C_4N_2CdI_4$ and $H_{11}C_4N_2CdI_3$.

Empirical formula	$C_4H_{12}CdI_4N_2$	$C_4H_{11}CdI_3N_2$
Formula weight	708.16	580.25
Temperature/K	293(2)	109(3)
Crystal system	orthorhombic	monoclinic
Space group	$P2_12_12_1$	*Cc*
a/Å	9.0318(5)	14.3680(6)
b/Å	12.2358(6)	7.13490(10)
c/Å	13.0518(7)	13.7778(5)
β/°	90	121.298(5)
Volume/Å^3	1442.37(13)	1206.88(9)
Z	4	4
ρ_{calcg}/cm^3	3.261	3.193
μ/mm^{-1}	10.037	74.345
F(000)	1240.0	1024.0
Radiation	Mo Kα (λ = 0.71073)	Cu Kα (λ = 1.54184)
Goodness-of-fit on F^2	1.047	1.061
Flack factor	0.45(12)	-0.01(2)
R_1, wR_2 [I >2σ(I)][a]	0.0408, 0.0850	0.0424, 0.1041
R_1, wR_2 (all data)[a]	0.0461, 0.0883	0.0425, 0.1043

[a]$R_1 = \sum||F_o| - |F_c||/\sum|F_o|$, and $wR_2 = \{\sum w[(F_o)^2 - (Fc)^2]^2/\sum w[(F_o)^2]^2\}^{1/2}$.

Table S3. Fractional Atomic Coordinates (× 10^4) and Equivalent Isotropic Displacement Parameters ($Å^2 \times 10^3$) for $H_{12}C_4N_2CdI_4$ and $H_{11}C_4N_2CdI_3$.

$H_{12}C_4N_2CdI_4$

Atom	x	y	z	U(eq)
Cd1	7352.6(12)	5392.0(8)	4092.0(8)	36.0(3)
I1	4716.6(11)	4175.1(8)	4038.7(8)	39.1(3)
I2	9616.5(10)	3847.3(7)	4066.0(8)	35.9(3)
I3	7585.9(14)	6743.7(8)	5772.2(8)	48.7(3)
I4	7413.3(11)	6482.7(7)	2231.4(7)	35.8(3)
N1	846(13)	5144(9)	1648(10)	34(3)
N2	4013(14)	5218(10)	1510(10)	42(3)
C1	1718(17)	4124(12)	1510(14)	43(4)
C2	3141(16)	4358(12)	960(14)	41(4)
C4	3116(17)	6228(13)	1640(14)	44(4)
C3	1713(17)	5982(11)	2195(12)	35(3)

$H_{11}C_4N_2CdI_3$

Atom	x	y	z	U(eq)
Cd1	4974.1(7)	6011.5(10)	4228.8(8)	23.2(2)
I1	6725.4(6)	8393.8(10)	5051.9(6)	26.5(2)
I2	4722.2(6)	3434.9(10)	2623.2(6)	26.0(2)
I3	3121.0(6)	7765.4(10)	3858.2(7)	27.4(2)
C1	6866(12)	2200(20)	7340(13)	33(3)
C2	6582(12)	3394(19)	6334(14)	30(3)
C3	4919(11)	1270(19)	6452(11)	25(3)
C4	4679(12)	2531(19)	5449(12)	26(3)
N1	6073(10)	593(17)	7009(10)	27(2)
N2	5449(9)	4080(13)	5754(10)	24(2)

Table S4. Hydrogen Atom Coordinates (Å×10^4) and Isotropic Displacement Parameters (Å^2×10^3) for $H_{12}C_4N_2CdI_4$ and $H_{11}C_4N_2CdI_3$.

$H_{12}C_4N_2CdI_4$

Atom	x	y	z	U(eq)
H2A	4285.58	4967.43	2121.84	50
H2B	4827.95	5371.16	1155.15	50
H1A	577.52	5401.06	1037.72	41
H1B	27.07	4996.09	2001.19	41
H1C	1142.15	3600.43	1118.83	51
H1D	1930.64	3804.86	2173.93	51
H2C	3722.95	3693.73	915.09	50
H2D	2927.61	4603.22	269.26	50
H4A	2889.96	6535.43	973.28	53
H4B	3682.14	6764.42	2022.68	53
H3A	1939.91	5725.27	2880.03	42
H3B	1129.68	6644.89	2256.11	42

$H_{11}C_4N_2CdI_3$

Atom	x	y	z	U(eq)
H1A	6140.14	-179.11	6523.12	32
H1B	6239.12	-80.51	7640.11	32
H2	5387.12	4854.1	6323.04	29
H4A	4692.35	1758.85	4859.07	32
H4B	3935.5	3051.89	5114.66	32
H1C	6851.68	2965.42	7930.59	39
H1D	7612.59	1696.33	7663.65	39
H3A	4814.1	1980.92	7005.48	30
H3B	4413.76	188.19	6181.75	30
H2A	7083.14	4480.4	6584.11	35
H2B	6689.26	2660.95	5789.62	35

Table S5. Bond Lengths for $H_{12}C_4N_2CdI_4$ and $H_{11}C_4N_2CdI_3$.

$H_{12}C_4N_2CdI_4$

Atom	Atom	Length/Å	Atom	Atom	Length/Å
Cd1	I1	2.8089(14)	N2	C4	1.488(19)
Cd1	I2	2.7847(14)	N1	C1	1.487(18)
Cd1	I3	2.7548(14)	N1	C3	1.474(17)
Cd1	I4	2.7714(13)	C1	C2	1.50(2)
N2	C2	1.496(18)	C4	C3	1.49(2)

$H_{11}C_4N_2CdI_3$

Atom	Atom	Length/Å	Atom	Atom	Length/Å
Cd1	I2	2.7518(11)	N1	C3	1.500(18)
Cd1	I1	2.7463(11)	N2	C4	1.462(17)
Cd1	I3	2.7393(12)	N2	C2	1.476(18)
Cd1	N2	2.300(11)	C4	C3	1.532(17)
N1	C1	1.509(19)	C1	C2	1.49(2)

Table S6. Bond Angles for $H_{12}C_4N_2CdI_4$ and $H_{11}C_4N_2CdI_3$.

$H_{12}C_4N_2CdI_4$

Atom	Atom	Atom	Angle/°	Atom	Atom	Atom	Angle/°
I4	Cd1	I2	107.56(5)	C4	N2	C2	110.6(11)
I4	Cd1	I1	104.50(4)	C3	N1	C1	111.2(11)
I2	Cd1	I1	105.21(4)	N1	C1	C2	110.6(12)
I3	Cd1	I4	114.01(4)	N2	C2	C1	110.9(13)
I3	Cd1	I2	111.16(5)	N2	C4	C3	110.5(12)
I3	Cd1	I1	113.75(5)	N1	C3	C4	110.9(12)

$H_{11}C_4N_2CdI_3$

Atom	Atom	Atom	Angle/°	Atom	Atom	Atom	Angle/°
I1	Cd1	I2	117.07(4)	C4	N2	Cd1	111.7(8)
I3	Cd1	I2	116.50(4)	C4	N2	C2	111.2(10)
I3	Cd1	I1	112.80(3)	C2	N2	Cd1	114.5(9)
N2	Cd1	I2	100.8(3)	N2	C4	C3	113.7(11)
N2	Cd1	I1	102.7(3)	C2	C1	N1	110.7(11)
N2	Cd1	I3	104.0(3)	N1	C3	C4	108.7(11)
C3	N1	C1	111.8(11)	N2	C2	C1	112.7(13)

Table S7. Theoretical and experimental results of elemental analysis for $H_{12}C_4N_2CdI_4$ and $H_{11}C_4N_2CdI_3$.

Weight (%)	$H_{11}C_4N_2CdI_3$ (Exp.)	$H_{11}C_4N_2CdI_3$ (Cal.)	$H_{12}C_4N_2CdI_4$ (Exp.)	$H_{12}C_4N_2CdI_4$ (Cal.)
C	8.30	8.27	6.77	6.77
H	1.85	1.9	1.63	1.69
N	4.53	4.82	4.02	3.95
Ratio				
C	4.01	4	3.99	4
H	10.73	11	11.54	12
N	2.19	2	2.37	2

Table S8. The assignments of the infrared absorption peaks for $H_{12}C_4N_2CdI_4$ and $H_{11}C_4N_2CdI_3$.

Assignment (cm^{-1})	$H_{12}C_4N_2CdI_4$	$H_{11}C_4N_2CdI_3$
ν(N-H)	3424, 3069	3424, 3079
ν(C-H)	3007, 2779	3003, 2776
ν(C-N)	1535	1541
ν(C-C)	1451	1456
ν(Cd-I)	544	549

Table S9. The convergence test of the SHG coefficient upon k-point sampling and empty bands of $H_{11}C_4N_2CdI_3$**.**

$H_{11}C_4N_2CdI_3$	The largest SHG tensor d_{11}(pm/V)			
k-point sampling ($Å^{-1}$)	Empty bands			
	1 * VB (140)	1.5 * VB (210)	2 * VB (280)	3 * VB (420)
k = 0.07, 0.06 (1*2*1)	-3.713	-3.215	-3.153	-3.158
k = 0.05, 0.04 (2*3*2)	-3.214	-2.821	-2.742	-2.742
k = 0.03 (3*4*3)	-3.105	-2.709	-2.642	-2.639

Table S10. Calculated dipole moment for CdI_4, $CdNI_3$ octahedra and net dipole moment for a unit cell in $H_{12}C_4N_2CdI_4$ and $H_{11}C_4N_2CdI_3$.

$H_{12}C_4N_2CdI_4$ (Z=4)				
Polar unit	Dipole moment (D=Debyes)			
	x-component	y-component	z-component	Total magnitude
$Cd(1)I_4$	-0.27856	-2.37235	-2.24159	3.275726
$Cd(2)I_4$	0.27453	2.367217	-2.24446	3.273636
$Cd(3)I_4$	-0.28012	2.365702	2.236867	3.267811
$Cd(4)I_4$	0.275477	-2.36971	2.238542	3.271463
Net dipole moment (a unit cell)	0	0	0	0
$H_{11}C_4N_2CdI_3$ (Z=4)				
Polar unit	Dipole moment (D=Debyes)			
	x-component	y-component	z-component	Total magnitude
$Cd(1)NI_3$	-4.15922	-3.83444	0.969529	5.739514
$Cd(2)NI_3$	-4.16198	-3.83499	0.969192	5.741825
$Cd(3)NI_3$	-4.16133	3.830677	0.966309	5.73799
$Cd(4)NI_3$	-4.16327	3.830389	0.968833	5.73963
Net dipole moment (a unit cell)	-16.6458	0	3.873863	17.09063

Table S11. SHG response and energy bandgap of representative NLO organic-inorganic halides.

Compound	Structural feature	SHG response	Band gap (eV)	Ref.
(L/D-$C_5H_{11}NO_3$)$PbI_3 \cdot 3H_2O$	With planar π-conjugate groups and SCALP cation	9.2×KDP	2.9	1
$Cs_3Pb_2(CH_3COO)_2X_5$ (X=I, Br)	With planar π-conjugate groups and SCALP cation	8, 4×KDP	2.55, 3.26	2
$Cs_3Pb_2(CH_3COO)_2Br_3I_2$	With planar π-conjugate groups and SCALP cation	9×KDP	2.70	3
$A_2[PbI_2(HCOO)_2]$ (A=K, Rb)	With planar π-conjugate groups and SCALP cation	8, 6.8×KDP	3.36, 3.40	4
$(C_6H_{11}N_2)PbBr_3$	With planar π-conjugate groups and SCALP cation	8×KDP	3.53	5
$(C_{18}H_{21}N_4)AgX_4$(X=Cl, Br, I)	With planar π-conjugate groups	6.2, 6.5, 7.6×KDP	3.26, 3.08, 2.63	6
$[N(CH_3)_4]HgCl_{0.63}Br_{2.37}$	Without planar π-conjugate groups and SCALP cation	0.25×KDP	3.62	7
$[N(CH_3)_4]HgBrI_2$	Without planar π-conjugate groups and SCALP cation	4.5×KDP	2.83	7
$[N(CH_3)_4]HgCl_{0.45}I_{2.55}$	Without planar π-conjugate groups and SCALP cation	6.2×KDP	2.76	7
$KCs_2[Pb_2Br_5(HCOO)_2]$	With planar π-conjugate groups and SCALP cation	6.5×KDP	3.23	8
$K_2I[PbI(OOCCH_2COO)]$	With planar π-conjugate groups and SCALP cation	6.3×KDP	3.34	9
$Rb_3Pb_2(CH_3COO)_2X_5$ (X = Br, Cl)	With planar π-conjugate groups and SCALP cation	6, 3×KDP	3.12, 3.64	10
$CH_3NH_3GeBr_3$	With SCALP cation	5.3×KDP	2.91	11
$CH(NH_2)_2GeBr_3$	With SCALP cation	0.9×KDP	3.13	11
$(CH_3NH_3)_{0.5}(CH(NH_2)_2)_{0.5}GeBr_3$	With SCALP cation	1.95×KDP	3.02	11
$KCs_2Pb_2(HCOO)_2Cl_5$	With planar π-conjugate groups and SCALP cation	4.2×KDP	3.52	12
$APb_2(C_7H_3NO_4)_2I$ (A=K, Rb, Cs)	With planar π-conjugate groups and SCALP cation	3.4, 1.6, 2.4×KDP	3.05, 3.05, 3.06	13
$(C_6H_{13}NCl)SbX_4$(X=Cl, Br)	With SCALP cation	1.8, 3.2×KDP	3.33, 2.82	14
$(C_3N_6H_7)(C_3N_6H_6)HgCl_3$	With planar π-conjugate groups	5×KDP	4.4	15
$(H_7C_3N_6)(H_6C_3N_6)ZnCl_3$	With planar π-conjugate groups	2.8×KDP	3.95	16
(R/S-$C_5H_{14}N_2$)PbI_4	With SCALP cation	2.1×KDP	2.94	17
(R/S-$C_5H_{14}N_2$)$SbCl_5$	With SCALP cation	1.93×KDP	3.06	18
$(C_9H_{14}N)SbCl_4$	With planar π-conjugate	2.1×KDP	3.47	19

	groups and SCALP cation			
$A_2Sb(C_2O_4)Cl_3$ (A=NH_4, K, Rb)	With planar π-conjugate groups and SCALP cation	1.8, 1.6, 2.1×KDP	3.55, 3.61, 3.74	20
$KPb_3(o\text{-}C_5H_4NCOO)_2Cl_5$	With planar π-conjugate groups and SCALP cation	2×KDP	3.79	21
α-$(CN_3H_6)_3Cu_2I_5$	With planar π-conjugate groups	1.8×KDP	2.80	22
(R/S-$C_6H_{14}N$)$PbBr_3$	With SCALP cation	1.4×KDP	3.51	23
$(C_{10}H_{14}N)PbBr_3$	With planar π-conjugate groups and SCALP cation	1.05×KDP	2.99	24
$(C_4H_{10}NO)PbX_3$(X=Cl, Br)	With SCALP cation	0.7, 0.81×KDP	3.55, 3.60	25
$[N(CH_3)_4]_2HgBr_2I_2$	Without planar π-conjugate groups and SCALP cation	0.46×KDP	2.8	26
$[N(CH_3)_4]_2HgI_4$	Without planar π-conjugate groups and SCALP cation	0.5×KDP	2.73	26
$[(CH_3)_3N]_3Bi_2I_9$	With SCALP cation	0.65×KDP	2.0	27
$(C_7H_{15}NCl)SbCl_4$	With SCALP cation	0.53×KDP	3.05	28
$(C_6H_5(CH_2)_4NH_3)_4BiBr_7\cdot H_2O$	With planar π-conjugate groups and SCALP cation	0.4×KDP	3.52	29
$(C_4H_{10}NO)_2Cd_2Cl_6$	Without planar π-conjugate groups and SCALP cation	0.73×KDP	5.45	30
$[(CH_3)_3NCH_2Cl]CdCl_3$	Without planar π-conjugate groups and SCALP cation	0.73×KDP	5.24	31
$[C_5H_{14}NO]CdCl_3$	Without planar π-conjugate groups and SCALP cation	0.4×KDP	4.41	32
L/D-$C_6H_{10}N_3O_2ZnBr_3$	With planar π-conjugate groups	0.2×KDP	5.02, 5.02	33
L/D-$C_{12}H_{20}N_6O_4Cd_2Cl_5$	With planar π-conjugate groups	0.2×KDP	5.01, 4.97	33
(L/D-$C_{10}H_{20}N_2O_4$)Cd_5Cl_{12}	With planar π-conjugate groups	0.25, 0.3×KDP	5.42, 5.42	34
(L/D-$C_{10}H_{19}N_2O_4$)$CdCl_3$	With planar π-conjugate groups	0.69, 0.71×KDP	5.63, 5.36	34
$(C_{20}H_{20}P)CuX_2$(X=Cl, Br)	With planar π-conjugate groups	1.1, 0.89×KDP	3.56,3.64	35
$H_{11}C_4N_2CdI_3$	Without planar π-conjugate groups and SCALP cation	6×KDP	4.10	**This work**

Table S12. The calculated SHG coefficients

compounds	SHG tensors d_{ij} (pm/V)
$H_{11}C_4N_2CdI_3$	d_{11} = -2.74<br>d_{12} = d_{26} = 0.94<br>d_{13} = d_{35} = 0.65<br>d_{15} = d_{31} = -0.55<br>d_{24} = d_{32} = -0.60<br>d_{33} = -1.22
$H_{12}C_4N_2CdI_4$	d_{14} = d_{25} = d_{36} = 0.12

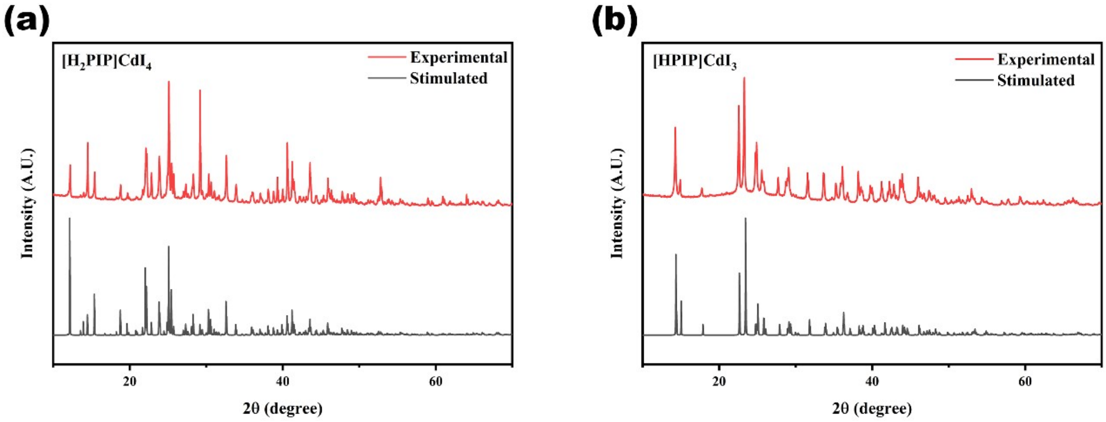

Figure S1. Simulated and measured powder X-ray diffraction patterns of (a) $H_{12}C_4N_2CdI_4$ and (b) $H_{11}C_4N_2CdI_3$.

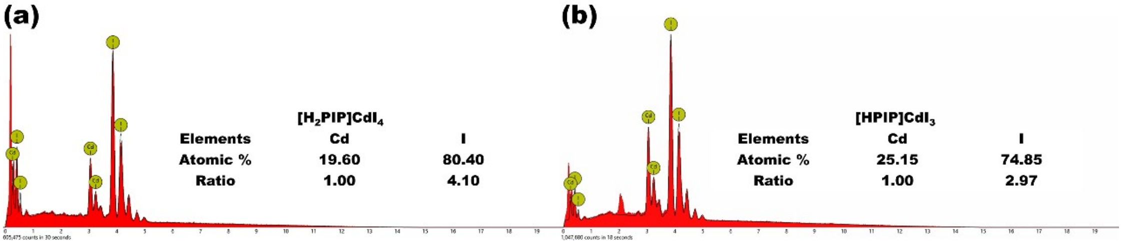

Figure S2. EDS for (a) $H_{12}C_4N_2CdI_4$ and (b) $H_{11}C_4N_2CdI_3$

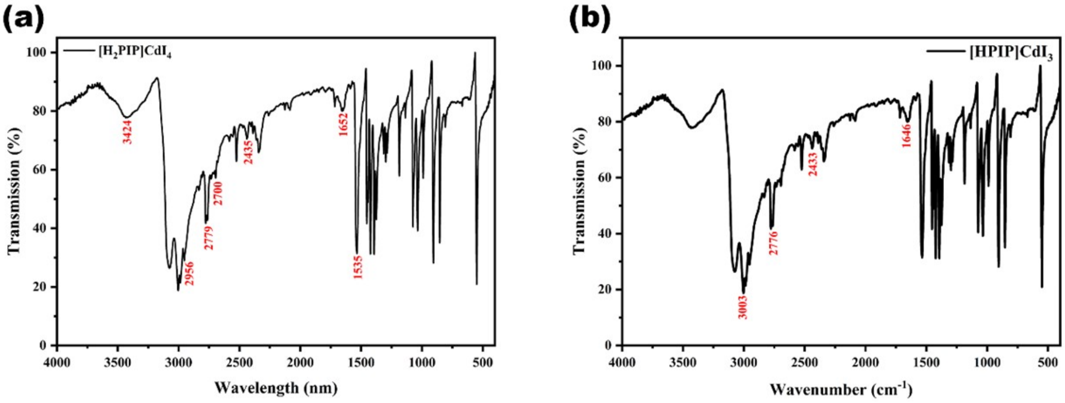

Figure S3. IR spectra of (a) $H_{12}C_4N_2CdI_4$ and (b) $H_{11}C_4N_2CdI_3$.

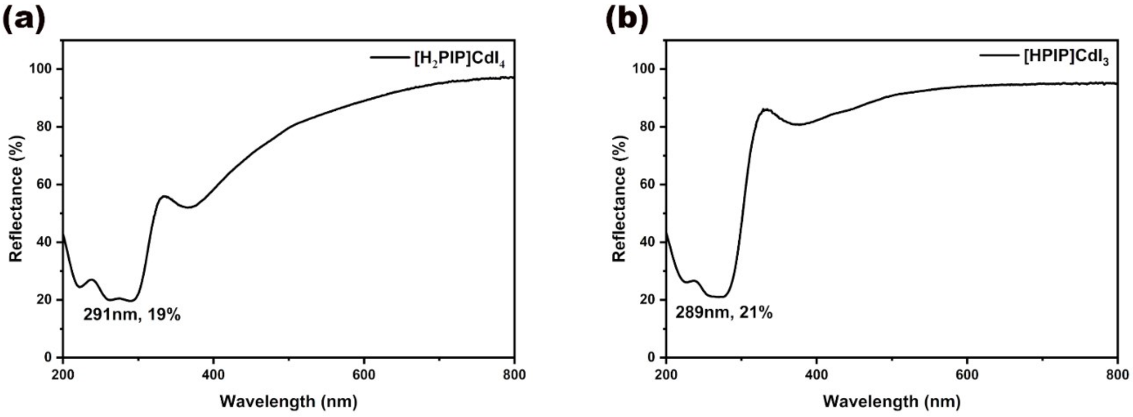

Figure S4. UV−vis−IR spectra of (a) $H_{12}C_4N_2CdI_4$ and (b) $H_{11}C_4N_2CdI_3$.

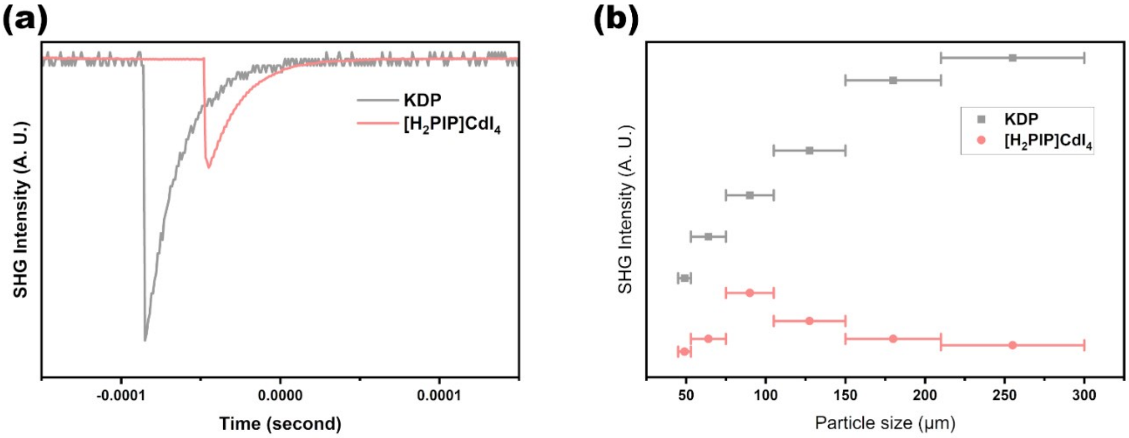

Figure S5. The SHG signal of $H_{12}C_4N_2CdI_4$ and SHG intensity vs. particle size of compounds under 1064 nm laser radiation of $H_{12}C_4N_2CdI_4$.

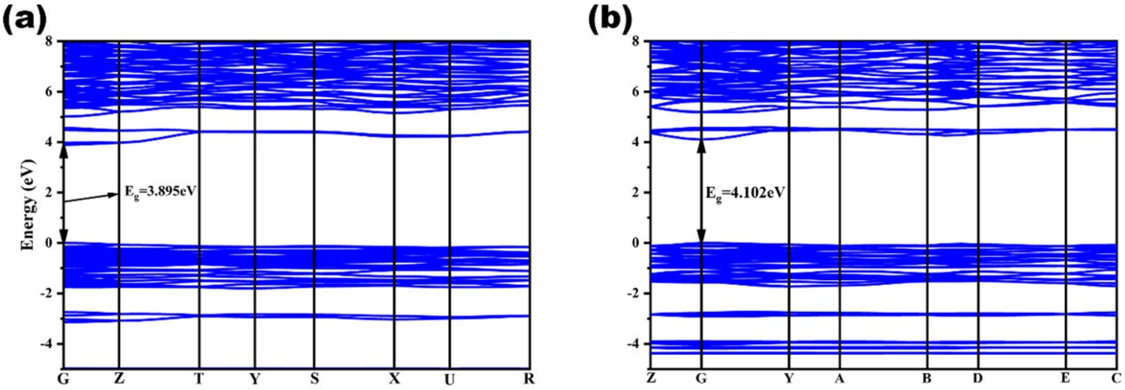

Figure S6. The calculated band structures of (a) $H_{12}C_4N_2CdI_4$ and (b) $H_{11}C_4N_2CdI_3$.

References

1. M. Xin, P. Cheng, X. Han, R. Shi, Y. Zheng, J. Guan, H. Chen, C. Wang, Y. Liu, J. Xu and X. H. Bu, Resonant Second Harmonic Generation in Proline Hybrid Lead Halide Perovskites, *Adv. Optical Mater.* , 2023, **11**, 2202700.
2. Q. R. Shui, H. X. Tang, R. B. Fu, Y. B. Fang, Z. J. Ma and X. T. Wu, $Cs_3Pb_2(CH_3COO)_2X_5$ (X=I, Br): Halides with Strong Second-Harmonic Generation Response Induced by Acetate Groups, *Angew. Chem. Int. Ed.* , 2021, **60**, 2116-2119.
3. Q. R. Shui, R. B. Fu, Z. Q. Zhou, Z. J. Ma, H. X. Tang and X. T. Wu, A Lead Mixed Halide with Three Different Coordinated Anions and Strong Second-Harmonic Generation Response, *Chem. Eur. J.* , 2022, **28**, e202103687.
4. X. Y. Zhang, Z. Q. Zhou, W. X. Bao, H. X. Tang, R. B. Fu, Z. J. Ma and X. T. Wu, New lead-iodide formates with a strong second-harmonic generation response and suitable birefringence obtained by the substitution strategy, *J. Name.*, 2022, **14**, 136-142.
5. Y. Deng, X. Dong, M. Yang, H. Zeng, G. Zou and Z. Lin, Two low-dimensional metal halides: ionothermal synthesis, photoluminescence, and nonlinear optical properties, *Dalton Trans.*, 2019, **48**, 17451-17455.
6. X. Y. Li, Q. Wei, C. L. Hu, J. Pan, B. X. Li, Z. Z. Xue, X. Y. Li, J. H. Li, J. G. Mao and G. M. Wang, Achieving Large Second Harmonic Generation Effects via Optimal Planar Alignment of Triangular Units, *Adv. Funct. Mater.* , 2022, **33**, 2210718.
7. C. Yang, X. Liu, C. Teng, Q. Wu and F. Liang, Syntheses, structure and properties of a new series of organic-inorganic Hg-based halides: adjusting halogens resulted in huge performance mutations, *Dalton Trans.*, 2021, **50**, 7563-7570.
8. Z. Q. Zhou, Q. R. Shui, R. B. Fu, Y. B. Fang, Z. J. Ma and X. T. Wu, $KCs_2[Pb_2Br_5(HCOO)_2]$: A Polar 3D Lead-Bromide Framework Exhibiting Strong Second-Harmonic Generation Response, *Chem. Eur. J.*, 2021, **27**, 12976-12980.
9. Z.-Q. Zhou, R.-B. Fu, H.-X. Tang, Z.-J. Ma and X.-T. Wu, An excellent lead oxyiodide with a strong second-harmonic generation response and a large birefringence induced by the oriented arrangement of highly distorted $[PbO_4I_2]$ polyhedra, *Inorg. Chem. Front.*, 2022, **9**, 4464-4469.
10. Q. Shui, R. Fu, H. Tang, Y. Fang, Z. Ma and X. Wu, Two Lead Halides with Strong SHG Response Obtained by the Isovalent Substitutions of Alkali Metal Cation and Halogen Anion, *Inorg. Chem.* , 2021, **60**, 5290-5296.
11. Y. Liu, Y. P. Gong, S. Geng, M. L. Feng, D. Manidaki, Z. Deng, C. C. Stoumpos, P. Canepa, Z. Xiao, W. X. Zhang and L. Mao, Hybrid Germanium Bromide Perovskites with Tunable Second Harmonic Generation, *Angew. Chem. Int. Ed.* , 2022, **61**, e202208875.
12. Z. Zhou, R. Fu, Q. Shui, H. Tang, W. Bao, Z. Ma and X. Wu, $KCs_2Pb_2(HCOO)_2Cl_5$: A Lead Formate with Strong Second-Harmonic-

Generation Response Obtained by an Anionic Substitution, *Inorg. Chem.* , 2022, **61**, 1130-1135.
13. Z.-L. Geng, Z.-Q. Zhou, H.-X. Tang, W.-X. Bao, R.-B. Fu and X.-T. Wu, $APb_2(C_7H_3NO_4)_2I$ (A = K, Rb, Cs): rare stable nonlinear optical crystals with second-harmonic generation response and highly distorted lead core coordination polyhedra, *Inorg. Chem. Front.*, 2022, **9**, 5783-5787.
14. Z. Qi, Y. Chen, H. Gao, F.-Q. Zhang, S.-L. Li and X.-M. Zhang, Two SbX_5-based isostructural polar 1D hybrid antimony halides with tunable broadband emission, nonlinear optics, and semiconductor properties, *Sci China Chem.* , 2021, **64**, 2111-2117.
15. Z. Bai, J. Lee, H. Kim, C. L. Hu and K. M. Ok, Unveiling the Superior Optical Properties of Novel Melamine-Based Nonlinear Optical Material with Strong Second-Harmonic Generation and Giant Optical Anisotropy, *Small.*, 2023, DOI: 10.1002/smll.202301756, e2301756.
16. L. Liu, Z. Bai, L. Hu, D. Wei, Z. Lin and L. Zhang, A melamine-based organic–inorganic hybrid material revealing excellent optical performance and moderate thermal stability, *J. Mater. Chem. C.*, 2021, **9**, 7452-7457.
17. D. Fu, J. Xin, Y. He, S. Wu, X. Zhang, X. M. Zhang and J. Luo, Chirality-Dependent Second-Order Nonlinear Optical Effect in 1D Organic-Inorganic Hybrid Perovskite Bulk Single Crystal, *Angew. Chem. Int. Ed.* , 2021, **60**, 20021-20026.
18. S. Qi, P. Cheng, X. Han, F. Ge, R. Shi, L. Xu, G. Li and J. Xu, Organic–Inorganic Hybrid Antimony(III) Halides for Second Harmonic Generation, *Cryst. Growth Des.* , 2022, **22**, 6545-6553.
19. F. Wu, Q. Wei, X. Li, Y. Liu, W. Huang, Q. Chen, B. Li, J. Luo and X. Liu, Cooperative Enhancement of Second Harmonic Generation in an Organic–Inorganic Hybrid Antimony Halide, *Cryst. Growth Des.* , 2022, **22**, 3875-3881.
20. D. Zhang, Q. Wang, H. Luo, L. Cao, X. Dong, L. Huang, D. Gao and G. Zou, Deep Eutectic Solvents Synthesis of $A_2Sb(C_2O_4)Cl_3$ (A = NH_4, K, Rb) with Superior Optical Performance, *Adv. Optical Mater.* , 2023, **11**.
21. W.-X. Bao, Z.-Q. Zhou, H.-X. Tang, R.-B. Fu, Z.-J. Ma and X.-T. Wu, $KPb_3(o\text{-}C_5H_4NCOO)_2Cl_5$: a brand-new stable lead chloride with good comprehensive nonlinear optical performance, *Inorg. Chem. Front.*, 2022, **9**, 1830-1835.
22. J. Wu, Y. Guo, J. L. Qi, W. D. Yao, S. X. Yu, W. Liu and S. P. Guo, Multi-Stimuli Responsive Luminescence and Domino Phase Transition of Hybrid Copper Halides with Nonlinear Optical Switching Behavior, *Angew. Chem. Int. Ed.* , 2023, **62**, e202301937.
23. Y. Zheng, J. Xu and X. H. Bu, 1D Chiral Lead Halide Perovskites with Superior Second‐Order Optical Nonlinearity, *Adv. Optical Mater.* , 2021, **10**.
24. K. Li, H. Ye, X. Li, X. Wang, J. Luo and X. Liu, Rational design of an organic–inorganic hybrid with Schiff base cations for an efficient quadratic nonlinear optical switch, *Inorg. Chem. Front.* , 2023, **10**, 435-442.

25. C. Shen, D. Sun, Y. Dang, K. Wu, T. Xu, R. Hou, H. Chen, J. Wang and D. Wang, $(C_4H_{10}NO)PbX_3$ (X = Cl, Br): Design of Two Lead Halide Perovskite Crystals with Moderate Nonlinear Optical Properties, *Inorg. Chem.* , 2022, **61**, 16936-16943.
26. C. Yang, X. Liu, C. Teng, Q. Wu and F. Liang, Acentric Organic-Inorganic Hybrid Halide $[N(CH_3)_4]_2HgBr_2I_2$ Featuring an Isolated $[HgBr_2I_2]^{2-}$Tetrahedron and Second-Order Nonlinearity, *Inorg. Chem.* , 2021, **60**, 6829-6835.
27. J. Zhang, S. Han, C. Ji, W. Zhang, Y. Wang, K. Tao, Z. Sun and J. Luo, $[(CH_3)_3NH]_3Bi_2I_9$: A Polar Lead-Free Hybrid Perovskite-Like Material as a Potential Semiconducting Absorber, *Chem. Eur. J.* , 2017, **23**, 17304-17310.
28. J.-M. Gong, T. Shao, P.-Z. Huang, C.-Y. Su, M. Chen, D.-W. Fu and H.-F. Lu, Reversible Phase Transition and Second-Harmonic Response Based on a Zero-Dimensional Organic–Inorganic Hybrid Compound, *J. Phys. Chem. C.*, 2022, **126**, 15274-15279.
29. D. Chen, S. Hao, L. Fan, Y. Guo, J. Yao, C. Wolverton, M. G. Kanatzidis, J. Zhao and Q. Liu, Broad Photoluminescence and Second-Harmonic Generation in the Noncentrosymmetric Organic–Inorganic Hybrid Halide $(C_6H_5(CH_2)_4NH_3)_4MX_7{\cdot}H_2O$ (M = Bi, In, X = Br or I), *Chem. Mater.* , 2021, **33**, 8106-8111.
30. D. Sun, D. Wang, Y. Dang, S. Zhang, H. Chen, R. Hou, K. Wu and C. Shen, Organic-Inorganic Hybrid Noncentrosymmetric $(Morpholinium)_2Cd_2Cl_6$ Single Crystals: Synthesis, Nonlinear Optical Properties, and Stability, *Inorg. Chem.* , 2022, **61**, 8076-8082.
31. C. Shen, J. Liu, K. Wu, L. Xu, D. Sun, Y. Dang, J. Wang and D. Wang, High stability and moderate second-order nonlinear optical properties of hybrid lead-free perovskite $[(CH_3)_3NCH_2Cl]CdCl_3$ bulk crystals, *J. Name.* , 2023, **25**, 2264-2270.
32. H. Cheng, C. Cao, S. Teng, Z. Zhang, Y. Zhang, D. Wang, W. Yang and R. Xie, Sn(II)-doped one-dimensional hybrid metal halide $[C_5H_{14}NO]CdCl_3$ single crystals with broadband greenish-yellow light emission, *Dalton Trans.* , 2023, **52**, 1021-1029.
33. W. Seo and K. M. Ok, Novel noncentrosymmetric polar coordination compounds derived from chiral histidine ligands, *Inorg. Chem. Front.* , 2021, **8**, 4536-4543.
34. J. Cheng, Y. Deng, X. Dong, J. Li, L. Huang, H. Zeng, G. Zou and Z. Lin, Homochiral Hybrid Organic-Inorganic Cadmium Chlorides Directed by Enantiopure Amino Acids, *Inorg. Chem.* , 2022, **61**, 11032-11035.
35. J.-L. Qi, J. Wu, Y. Guo, Z.-P. Xu, W. Liu and S.-P. Guo, Quasi-linear CuX_2 (X = Cl, Br) motif-built hybrid copper halides realizing encouraging nonlinear optical activities, *Inorg. Chem. Front.* , 2023, DOI: 10.1039/d3qi00297g.
